# Fintech investments in European banks: a hybrid IT2 fuzzy multidimensional decision-making approach

**DOI:** 10.1186/s40854-021-00256-y

**Published:** 2021-05-21

**Authors:** Gang Kou, Özlem Olgu Akdeniz, Hasan Dinçer, Serhat Yüksel

**Affiliations:** 1 School of Business Administration, Southwestern University of Finance and Economics, Chengdu, 611130 China; 2 College of Administrative Sciences and Economics, Koç University, Rumeli Feneri Yolu, Sarıyer, Istanbul, 34450 Turkey; 3 School of Business, Istanbul Medipol University, Kavacık Campus, Beykoz, Istanbul, 34810 Turkey

**Keywords:** Financial technology, European banking industry, Interval type-2 fuzzy TOPSIS, DEMATEL

## Abstract

Financial technology (Fintech) makes a significant contribution to the financial system by reducing costs, providing higher quality services and increasing customer satisfaction. Hence, new studies play an essential role to improve Fintech investments. This study evaluates Fintech-based investments of European banking services with an application of an original methodology that considers interval type-2 (IT2) fuzzy decision-making trial and evaluation laboratory and IT2 fuzzy TOPSIS models. Empirical findings are controlled for consistency by applying the VIKOR method. Moreover, we conduct a sensitivity analysis by considering six distinct cases. This study contributes to the existing literature by identifying the most important Fintech-based investment alternatives to improve the financial performance of European banks. Our empirical findings illustrate that results are coherent, reliable, and identify “competitive advantage” as the most important factor among Fintech-based determinants. Moreover, “payment and money transferring systems” are the most important Fintech-based investment alternatives. It is recommended that, among Fintech-based investments, European banks should mainly focus on payment and money transferring alternatives to attract the attention of customers and satisfy their expectations. This is also believed to have a positive impact on the ease of bank’ receivable collection. Another important point is that Fintech-based investments in money transferring systems could help to decrease costs.

## Introduction

Financial technology (Fintech) is the process of accomplishing technological investments to improve financial operations. It helps firms, including banks, to have a competitive advantage mainly by decreasing costs and increasing efficiency (Zhang and Yang 2019). Fintech is recognized as one of the most important innovations in the financial industry and is evolving at a rapid pace. Fintech promises to reshape the financial industry by cutting costs, improving the quality of financial services, and creating a more diverse and stable financial landscape (FinTech Revolution 2016). Moreover, the world has been battling the Covid 19 pandemic since the beginning of 2020, which is creating financial and psychological distress on sectors and economies. As a result, technology and innovation usage massively increased to eliminate the challenges caused by numerous precautions taken by governments such as local and/or national lockdowns. At this point, Fintech applications have stepped forward to catalyze businesses and individual processes. It provides important advantages such as strengthening businesses, processing big data to meaningful data, being universal, cheaper, and more secure compared to conventional methods (Lee and Shin 2018). In addition, Fintech eliminates traditional intermediaries while offering financial services (Thakor 2020). High operational costs are a challenging problem for all sectors exclusively for financial services. Since the beginning of the lockdown in Europe, the usage of Fintech applications has increased by 72 percent (Moden and Neufeld 2020). The recent global trends and the need to have quicker and cost-effective access to banking services has been the ultimate motivation of this research.

Moreover, developing global trends in international trade has had an important influence on the critical role of the global banking sector over recent years. Banks experience a challenge in terms of finding the right Fintech investments to increase their competitive power and satisfy demand of new customers in various countries (Cornaggia et al. 2015). The strategic location of Europe in terms of international trade can be suggested as one of the most important reasons for boosted competition in the European banking sector. With the purpose of handling this challenge, European banks pay an enhanced attention to research and development, customer satisfaction, new product development and organizational efficiency (Căpraru et al. 2020).

In this respect, we suggest numerous investment alternatives to enrich Fintech applications in banking. The first is increasing the effectiveness of money transferring systems (Shaikh et al. 2017). Banks can minimize the cost of money transfers, which in return provide an opportunity to increase sales volume. Furthermore, Fintech applications have a positive impact on bank payment systems as easy payment systems imply more effective collections of receivables on time (Eyal 2017).

The second alternative is related to savings. If essential technological investments are made, customers can have an opportunity to assess their savings easily (Ferrari 2016). Due to the user-friendly applications of these systems, customers may prefer to work with banks equipped with Fintech opportunities. Third, customers can manage their budget and take loans with less effort and time with the help of Fintech investments (Gozman et al. 2018; Liu et al. 2015). It can be suggested that these factors have a positive influence on the competitive power and organizational efficiency of banks.

Our focus is to assess suitable selection of Fintech-based investments in the European banking sector. In the first step, three financial and three non-financial criteria related to the advantages of Fintech-based investments are defined. Then, five Fintech-based investment alternatives are identified. An interval type-2 (IT2) fuzzy decision-making trial and evaluation laboratory (DEMATEL) is employed for weighting the criteria, followed by an application of an IT2 fuzzy TOPSIS model to rank the investment alternatives. Identifying the importance of Fintech-based investment alternatives helps provide suggestions for future research areas. We argue that payment and money transferring systems are the most important alternatives that play an important role in satisfying customer expectations, easing banks’ collection of receivables, and decreasing operational costs.

First, a hybrid model is considered, which means that different MCDM models are used in both weighting the criteria and ranking the alternatives. However, in non-hybrid models, only one MCDM technique is considered to rank the alternatives (Kumar et al. 2020). In this process, the researchers define the weights of the criteria (Mathew et al. 2020). We therefore suggest that hybrid methods have a positive contribution to the objectivity of the results (Yucesan and Gul 2020). In addition, examining the DEMATEL method in the analysis is another important novelty of this model. There are several approaches used to weight the criteria, such as the analytic hierarchy process (AHP), FUCOM, BWM, and level-based weight assessment (LBWA). However, the main superiority of the DEMATEL in comparison to others is the ability of generating the impact-relation map of the criteria (Xu et al. 2020; Garg 2021). This offers the opportunity to conduct causality evaluation among factors (Zhang et al. 2020a, b; Wang et al. 2020).

Another essential novelty of this proposed model is applying the TOPSIS method to rank the alternatives. Even though there are various methods such as MARCOS, VIKOR, and MABAC that can be considered for this purpose, we suggest the TOPSIS method is more suitable as it identifies distances to both positive and negative ideal solutions (Rani et al. 2020; Dhiman and Deb 2020). As a result, it is very helpful to reach more effective results (Rouyendegh et al. 2020; Ziemba et al. 2020). Moreover, considering IT2 fuzzy sets provides some benefits. To solve the decision-making problems more effectively, there is a strong need for a complex analysis to minimize the uncertainty in this process (Soto et al. 2019; Melin et al. 2012). Pulido et al. (2014) and Du et al. (2020) suggest that IT2 fuzzy sets have a positive influence to handle the uncertainty more effectively. Furthermore, we performed a consistency analysis using the VIKOR method to rank the alternatives, followed by a sensitivity analysis considering six individual cases. Hence, it can be possible to check the coherency and reliability of the empirical results. To the best of our knowledge, this study is the first application of a hybrid IT2 fuzzy multidimensional decision-making approach to identify the Fintech-based investment alternatives for European banks.

We believe the proposed model is relevant and serves the purpose of this study. In the first stage of the evaluation process, Fintech-based determinants are weighted. These factors can have an influence on each other. Instead of the AHP and the analytic network process (ANP), we selected the DAMATEL method as it can process a causality analysis between criteria. Additionally, Fintech-based investment alternatives for European banking services are ranked in the second stage of the analysis. As Fintech-based investment alternatives are crucial for improving the performance of the European banking industry, we carefully applied the TOPSIS and VIKOR approaches to rank the alternatives and for reliability analysis. The recommendations can pave the way for investors and policy makers.

The rest of the paper is organized as follows. “Literature review” section introduces the literature review, followed by the methodology in “Methodology” section. “Empirical findings” section introduces the empirical findings. Section 5 concludes, while highlighting and critically evaluating the empirical findings with suggestions to policymakers.

## Literature review

### Literature on Fintech

Fintech has become a popular research topic over the last decade, which has been evaluated in different aspects. For instance, Chen and Wu (2019) illustrated that it boosts effective consumer finance in China. Applying a SWOT analysis, the study suggested that Fintech applications have a significant influence on the credit system. Additionally, Zhou et al. (2018) provided supporting evidence for the positive impact of Fintech applications on the effectiveness of credit card systems. Sun (2018) and Chang et al. (2017) also underlined the importance of Fintech investments in the performance of such systems was also underlined.

Some studies focused on the importance of Fintech investment in blockchain systems. Nguyen (2016), Li et al. (2017), and Heiskanen (2017) concluded that Fintech and blockchain systems played a key role in sustainable economic development of countries. Treleaven et al. (2017) and Guo and Liang (2016) also determined that the operations of banking and finance can be simplified with the help of the blockchain system. Additionally, Guo and Liang (2016), Du et al. (2019), and Eyal (2017) investigated the importance of Fintech in banking. They indicated that the blockchain technology provides many advantages to the banking system, such as recording payment and credit information of customers.

Other studies emphasized the relationship between Fintech and payment systems of countries. Thompson (2017) outlined the advantages of mobile money application, which is explained as a new type of Fintech. The author revealed that Fintech mainly contributes to the effectiveness of the payment system. Similarly, Woldmariam et al. (2016) explored the design of Fintech in Ethiopia. In this framework, they evaluated mobile money applications. They argued that Fintech provides ease of operations for money payment systems. Additionally, Yao et al. (2018a, b), Shaikh et al. (2017), and Ramos-de-Luna et al. (2016) concluded that Fintech has a positive influence on companies’ payment systems.

In addition, Islamic Fintech is described as an important factor in the literature. For instance, Firmansyah and Anwar (2019) focused on Islamic Fintech and defined it as a new trend in Islamic finance. Via a survey analysis in Singapore and Indonesia, they concluded that investments in technology have a positive influence on the development of Islamic finance. Similarly, Bakar and Rosbi (2018) introduced a technical analysis from an Islamic Fintech perspective. They stated that Fintech investments attracted the attention of Islamic investors. Moreover, Rusydiana (2018) and Firmansyah and Ramdani (2018) suggested that technology-based investments lead to improvement in the Islamic financial system.

Some researchers have also discussed the impact of Fintech on customer satisfaction. Kabakova et al. (2016) analyzed Fintech development in Russia illustrating that it is a significant issue in customer satisfaction. In addition, Komulainen et al. (2018) applied an interview methodology to analyze the impact of Fintech investments on supply chain management. They concluded that Fintech is important to meet customer expectations. Furthermore, Xu and Cheng (2017), Yao et al. (2018a, b), Mittal et al. (2017), and Tan et al. (2018) found that Fintech investments are helpful to improve customers satisfaction.

In a different aspect, some researchers have examined the relationship between Fintech and competition in the market. Chen (2018) assessed Fintech applications in the USA and China. They concluded that Fintech investments are required to survive in competitive environments. In addition, Liu et al. (2015) determined that Fintech provides easiness in the use of payment systems, trading activities, and credit services. Therefore, it is argued that Fintech investments offer a competitive power for companies. Similarly, Kauffman et al. (2015), Kazan et al. (2018), and Gozman et al. (2018) proposed that companies should make Fintech investments to survive in a competitive environment.

Furthermore, reducing costs is another important outcome of Fintech investment. Zhang and Yang (2019) evaluated the Fintech system in China. They demonstrated that it has an important contribution for companies to decrease their costs and increase their profitability. Arner et al. (2018) also defined Fintech investment as the main source of decreasing costs. Similarly, Ko et al. (2018), Dula and Lee (2017), and Anderson et al. (2017) reached the same conclusion.

Lastly, Fintech is assessed for banking sectors. Nguyen (2016) and Guo and Liang (2016) underlined the importance of Fintech in the organizational efficiency of banks. Eyal (2017) also identified that Fintech plays a key role to increase market value. Moreover, Shaikh et al. (2017) and Ferrari (2016) identified Fintech investments as a significant way to increase sales volume of banks.

### Literature on MCDM models

Yu et al. (2021) introduced an excellent literature review on the AHP and ANP research with an application of bibliometric analysis over the 1982–2018 period. The study reviewed 9859 publications from Web of Science and illustrated important findings about the AHP/ANP research supported with future research suggestions. First, China is identified as the most productive country in terms of publications while the USA is the most highly cited country. Moreover, researchers in Malaysia, England, Iran, Australia, and the USA have the highest level of collaborations. From an institutional perspective, findings illustrated that institutions from China have the highest interest in the AHP/ANP studies, while from a regional perspective, AHP/ANP related publications in Asia have become popular in recent years.

Lin et al. (2020) also confirmed AHP as a commonly applied method in group decision-making and introduced an aggregated version of the nearest consistent matrices (ANCM). The authors emphasized advantages of the proposed model over other techniques and supported their discussion with empirical findings from two applications.

On the other hand, the DEMATEL and TOPSIS approaches have been applied by numerous researchers in different sectors. Xu et al. (2020) weighted the critical barriers to the development of hydrogen refueling stations in China by considering DEMATEL. Similarly, Feng and Ma (2020) identified the factors that have an influence on the service innovation in manufacturing enterprises using the fuzzy DEMATEL method. In addition, Farooque et al. (2020) used the methodology to analyze the barriers to Blockchain-based life cycle assessment in China. Moreover, Zhang et al. (2020a, b) investigated significant factors of youth unemployment using interval-valued intuitionistic hesitant fuzzy DEMATEL based on 2-tuple linguistic values. Zhong et al. (2020) and Yuan et al. (2020) focused on important strategies for renewable energy investments by applying DEMATEL.

Dogan et al. (2019) utilized TOPSIS regarding a corridor selection for locating autonomous vehicles. Furthermore, Demirel et al. (2016) introduced a comparative evaluation for location selection of textile plants in Turkey and ranked alternatives using TOPSIS. Deveci et al. (2020) and Türk et al. (2021) applied the methodology with IT2 fuzzy sets for offshore wind farm development and locating electric charging stations. In addition, Qiu et al. (2020) used TOPSIS to conduct risk evaluation of the wind energy investments in emerging economies. A recent study by Çalık (2021) applied Pythagorean fuzzy TOPSIS for green supplier selection in the era of Industry 4.0.

### Rationale of the study

It is possible to reach some conclusions after conducting this comprehensive review of the literature. First, the popularity of Fintech subjects has increased in the literature, especially in the last years. Many different researchers have focused on the advantages of this system for companies, such as cost minimization and customer satisfaction, by considering different industries. There is a need for new research that provides specific strategies to improve Fintech investments. Hence, this study evaluates Fintech-based investments for European banking services. The literature review also reveals that econometric models, such as regression and cointegration analysis, are considered in most of the studies regarding Fintech. The main limitation of these approaches is that only numerical indicators can be considered. Therefore, different methodologies that can consider both numerical and non-numerical determinants should be used. Hence, the current study proposes a novel model by considering IT2 fuzzy DEMATEL and IT2 fuzzy TOPSIS. These approaches have been used by various studies in the literature. In the current research, these approaches are used together to increase objectivity of the analysis results. Additionally, decision-making problems involve quite complex processes. These methods should also be developed to achieve more accurate and effective results. Thus, these methods are used with IT2 fuzzy sets to handle the uncertainties in this process more effectively.

## Methodology

**Fig. 1:**
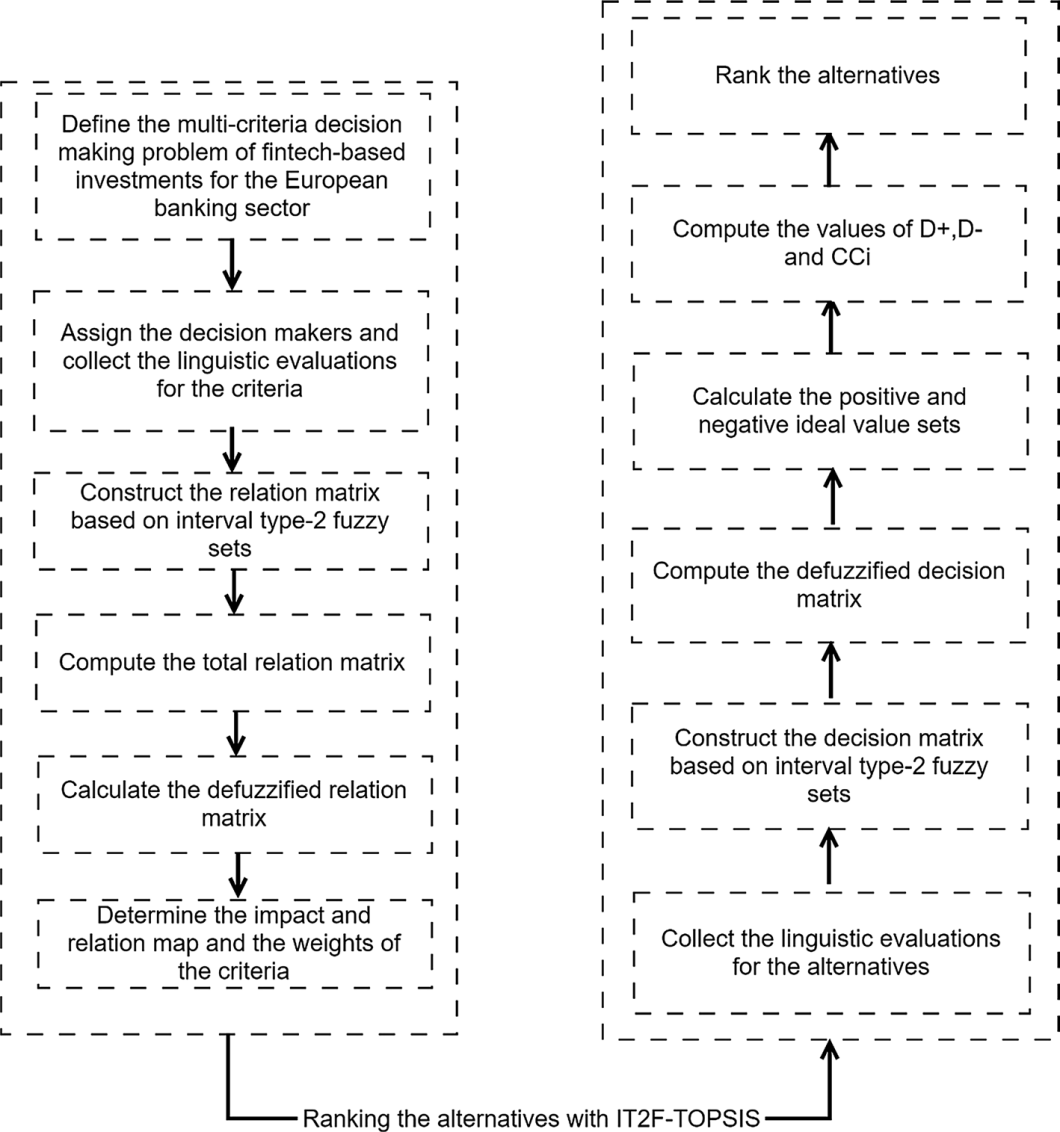
The flowchart of the proposed model

The model is estimated using an integrated decision-making approach based on IT2 fuzzy sets. IT2 fuzzy DEMATEL is employed to weight the Fintech-based determinants, followed by an application of the TOPSIS with IT2 fuzzy sets to rank the investment alternatives of the European banking sector. Additionally, a comparative analysis is performed using the fuzzy VIKOR method, followed by a sensitivity analysis. The details of the proposed model are illustrated in Fig. 1.

The stages and steps of this proposed model are highlighted as follows.

- Stage 1: Weighting the criteria with IT2F-DEMATEL
  - Step 1: Define the multi-criteria decision-making problem of Fintech-based investments for the European banking sector.
  - Step 2: Assign the decision-makers and collect the linguistic evaluations for the criteria.
  - Step 3: Construct the relation matrix based on IT2 fuzzy sets.
  - Step 4: Compute the total relation matrix.
  - Step 5: Calculate the defuzzified relation matrix.
  - Step 6: Determine the impact and relation map and the weights of the criteria.
- Stage 2: Ranking the alternatives with IT2F-TOPSIS
  - Step 7: Collect the linguistic evaluations for the alternatives.
  - Step 8: Construct the decision matrix based on IT2 fuzzy sets.
  - Step 9: Compute the defuzzified decision matrix.
  - Step 10: Calculate the positive and negative ideal value sets.
  - Step 11: Compute the values of D+ , D−, and CCi.
  - Step 12: Rank the alternatives.

The proposed model is a hybrid method that covers an application of the DEMATEL, TOPSIS, and VIKOR, and is suitable for the evaluation of Fintech-based investments of European banking services. It considers six selective criteria that may have an impact on each other. In addition to weighting the criteria, the cause-and-effect relationship of these items should also be investigated. With the help of the DEMATEL method, we generated an impact-relation map. In the final stage of our analysis, Fintech-based investment alternatives are ranked for the European banking services, which is crucial in proposing investment suggestions and policy implications. In this respect, both TOPSIS and VIKOR approaches are adopted to examine the consistency and coherency of the results.

### Data and variables

In Step 1 of our technical analysis, multi-criteria decision-making is defined to construct the model. We evaluated a set of criteria and alternatives based on the relevant literature. The selected criteria for problem definition are introduced in Table 1.

**Table 1:** Fintech-based determinants of the European banking sector. *Source*: Created by the authors

Dimensions	Criteria	References
Financial (Dimension 1)	Cost management (C1)	Zhang and Yang (2019), Arner et al. (2018), Ko et al. (2018), Dula and Lee (2017) and Anderson et al. (2017)
	Sales volume (C2)	Shaikh et al. (2017), Ferrari (2016) and Heiskanen (2017)
	Increase in market value (C3)	Eyal (2017), Zhou et al. (2018), Sun (2018) and Chang et al. (2017)
Non-financial (Dimension 2)	Customer satisfaction (C4)	Kabakova et al. (2016), Komulainen et al. (2018), Xu and Cheng (2017), Yao et al. (2018a, b), Mittal et al. (2017) and Tan et al. (2018)
	Competitive advantage (C5)	Chen (2018), Liu et al. (2015), Kauffman et al. (2015), Kazan et al. (2018) and Gozman et al. (2018)
	Organizational efficiency (C6)	Nguyen (2016), Guo and Liang (2016), Treleaven et al. (2017) and Guo and Liang (2016)

As shown in Table 1, three financial and three non-financial criteria are identified for Fintech-based investments. With respect to the financial criteria, cost management (C1) demonstrates that banks can undertake Fintech investment mainly to decrease operational costs as they can provide banking services at a lower cost. The next criterion, sales volume (C2), indicates that Fintech can have a positive influence on the sales volume. Additionally, increase in market value (C3) provides information that adopting this technology provides opportunity to have a greater market value.

Regarding non-financial dimensions, Fintech investments lead to higher customer satisfaction (C4) due to easily accessible and user-friendly operations. Another important point of Fintech investment is that it provides competitive advantage (C5) for banks. The final criterion, organizational efficiency (C6), explains that considering Fintech helps to improve communication among the departments. Our Fintech-based investment alternatives suggested for the European banking sector are presented in Table 2.

**Table 2:** Fintech-based investment alternatives for the European banking services. *Source*: Created by the authors

Alternatives	References
Money transferring (alternative 1)	Yao et al. (2018a, b), Shaikh et al. (2017) and Ramos-de-Luna et al. (2016)
Payments (alternative 2)	Guo and Liang (2016), Du et al. (2019) and Eyal (2017)
Savings (alternative 3)	Shaikh et al. (2017) and Ferrari (2016)
Budgeting (alternative 4)	Kazan et al. (2018) and Gozman et al. (2018)
Borrowings (alternative 5)	Chen (2018), Liu et al. (2015) and Kauffman et al. (2015)

Table 2 introduces our five suggested alternatives for Fintech investments. First, banks can make Fintech investments to money transferring systems (alternative 1). This is suggested as a cost cutting application as it removes the burden for customers traveling to a branch and helps save time. This is anticipated to increase banks’ sales volume and their revenues.

Second, banks can make Fintech investments to payment systems (alternative 2). If customers can pay their debt easily, banks would have a chance to collect their receivables on time. Third, if bank customers can easily estimate their savings (alternative 3) with the help of Fintech investments, they would prefer to work with these banks. Fourth, banks can make Fintech investments to improve budgeting operations (alternative 4), which can attract the attention of customers. Finally, Fintech investments can be directed to borrowing operations (alternative 5). If customers have easy access to and quick approval for a bank loan, this is expected to increase banks’ sales volume and revenues.

A set of criteria in two dimensions is evaluated by the expert team. A total of three decision-makers are appointed to provide their linguistic evaluations for the criteria and alternatives. Decision-makers are experts in the field of research and development in financial services with at least ten years’ experience. The details of the experts are reported in Table 3.

**Table 3:** The details decision makers (DM)

Decision makers	Level of education	Experience	Occupation
DM1	Ph.D.	29 years	Academic in banking, strategy development and risk management
DM2	Ph.D.	10 years	Academic in banking, finance, financial development
DM3	Ph.D.	22 years	Academic and CFO in a private bank

Linguistic evaluations for the criteria and alternatives are provided in Tables 4 and 5.

**Table 4:** Linguistic relation matrix for the criteria. *Source*: Author’s own table

	C1			C2			C3			C4			C5			C6		
	DM1	DM2	DM3	DM1	DM1	DM2	DM3	DM2	DM3	DM1	DM2	DM3	DM1	DM2	DM3	DM1	DM2	DM3
Cost management (C1)	–	–	–	H	H	MH	H	H	H	M	MH	MH	H	H	VH	H	VH	VH
Sales volume (C2)	M	ML	M	–	–	–	M	M	M	M	ML	M	ML	ML	ML	ML	ML	L
Increase in market value (C3)	ML	ML	L	M	M	M	–	–	–	ML	ML	M	M	MH	MH	M	H	MH
Customer satisfaction (C4)	M	M	M	M	MH	M	MH	MH	M	–	–	–	H	VH	H	VH	VH	H
Competitive advantage (C5)	M	MH	M	MH	MH	H	H	VH	H	M	MH	MH	–	–	–	H	VH	VH
Organizational efficiency (C6)	L	L	ML	ML	ML	L	ML	ML	M	L	ML	ML	M	ML	M	–	–	–

VH, very high; H, high; MH, medium high; M, medium; ML, medium low; L, low

**Table 5:** Linguistic decision matrix for the alternatives. *Source*: Author’s own table

	Money transferring (alternative 1)			Payments (alternative 2)			Savings (alternative 3)			Budgeting (alternative 4)			Borrowings (alternative 5)		
	DM1	DM2	DM3	DM1	DM1	DM2	DM3	DM2	DM3	DM1	DM2	DM3	DM1	DM2	DM3
Cost management (C1)	G	VG	B	VG	B	B	G	F	F	G	G	F	G	G	G
Sales volume (C2)	VG	G	VG	VG	B	VG	F	MP	MP	F	F	MP	G	F	F
Increase in market value (C3)	B	VG	G	B	VG	VG	F	F	F	G	F	G	G	G	VG
Customer satisfaction (C4)	VG	B	VG	B	B	VG	G	F	F	G	G	F	G	VG	VG
Competitive advantage (C5)	VG	VG	VG	VG	B	B	G	F	MP	F	F	F	G	G	G
Organizational efficiency (C6)	VG	B	B	B	VG	VG	MP	F	F	F	G	MP	F	F	G

VP, very poor; P, poor; MP, medium poor; F, fair; G, good, VG, very good; B, best

The values are converted into trapezoidal fuzzy numbers to analyze under the fuzzy environment. Linguistic scales and their IT2 fuzzy numbers are presented in “Appendix A”.

### Interval type-2 fuzzy sets

A type-2 fuzzy set is shown as $\tilde{A}$ while $\mu_{{\tilde{A}\left( {x,u} \right)}}$ gives information about the type-2 membership function. Details of this process are revealed in Eq. (1) (Soto et al. 2019; Zhou et al. 2020).

1 $$\tilde{A} = \left\{ {\left( {\left( {x,u} \right),\mu_{{\tilde{A}\left( {x,u} \right)}} } \right){|}\forall_{x} \in X,\forall_{u} \in J_{x} \subseteq \left[ {0,1} \right]} \right\},\,{\text{or}}\,\tilde{A} = \mathop \smallint \limits_{x \in X}^{{}} \mathop \smallint \limits_{{u \in J_{x} }}^{{}} \mu_{{\tilde{A}}} \left( {x,u} \right)/\left( {x,u} \right){ }J_{x} \subseteq \left[ {0,1} \right]$$

Within this context, $\mu_{{\tilde{A}}} \left( {x,u} \right)$ can take values between 0 and 1. Moreover, $\smallint \smallint$ identifies the union over all admissible *x* and *u*. Regarding the discrete universes, $\smallint$ can be replaced by ${\Sigma }$. When all $\mu_{{\tilde{A}}} \left( {x,u} \right)$ is equal to 1, $\tilde{A}$ can be shown ass in the Eq. (2) (Soto et al. 2018).

2 $$\tilde{A} = \mathop \smallint \limits_{x \in X}^{{}} \mathop \smallint \limits_{{u \in J_{x} }}^{{}} 1/\left( {x,u} \right){ }J_{x} \subseteq \left[ {0,1} \right]$$

**Fig. 2:**
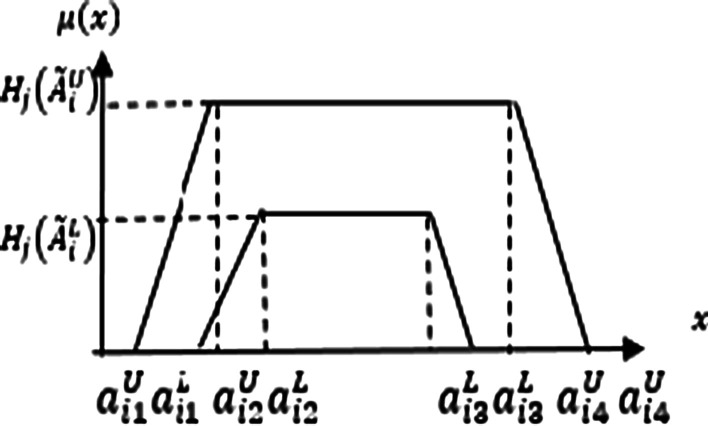
The trapezoidal membership function of the interval type-2 fuzzy set

Figure 2 provides information about the membership functions of IT2 fuzzy sets (Soto et al. 2014; Qiu et al. 2020).

In this scope, the upper trapezoidal membership function is represented by $\tilde{A}_{i}^{U}$. Additionally, $\tilde{A}_{i}^{L}$ identifies the lower trapezoidal membership function. The details are presented in Eq. (3) (Pulido et al. 2014; Dinçer et al. 2019).

3 $$\tilde{A}_{i} = \left( {\tilde{A}_{i}^{U} ,\tilde{A}_{i}^{L} } \right) = \left( {\left( {a_{i1}^{U} ,a_{i2}^{U} ,a_{i3}^{U} ,a_{i4}^{U} ;H_{1} \left( {\tilde{A}_{i}^{U} } \right),H_{2} \left( {\tilde{A}_{i}^{U} } \right)} \right),\left( {a_{i1}^{L} ,a_{i2}^{L} ,a_{i3}^{L} ,a_{i4}^{L} ;H_{1} \left( {\tilde{A}_{i}^{L} } \right),H_{2} \left( {\tilde{A}_{i}^{L} } \right)} \right)} \right)$$

In this equation, $a_{i1}^{U} ,a_{i2}^{U} ,a_{i3}^{U} ,a_{i4}^{U}$, ${ }a_{i1}^{L} ,a_{i2}^{L} ,a_{i3}^{L} ,a_{i4}^{L}$ are the reference values of the IT2 fuzzy set. $H_{j} \left( {\tilde{A}_{i}^{U} } \right)$ represents the membership value in the upper trapezoidal membership function whereas $H_{j} \left( {\tilde{A}_{i}^{L} } \right)$ shows that in the lower trapezoidal membership function. Details are given in Eqs. (4) –(8) (Melin et al. 2012; Du et al. 2020).

4 $$\begin{aligned} \tilde{A}_{1} \oplus \tilde{A}_{2} & = \left( {\tilde{A}_{1}^{U} ,\tilde{A}_{1}^{L} } \right) \oplus \left( {\tilde{A}_{2}^{U} ,\tilde{A}_{2}^{L} } \right) = \left( {\left( {a_{11}^{U} + a_{21}^{U} ,a_{12}^{U} + a_{22}^{U} ,a_{13}^{U} + a_{23}^{U} ,a_{14}^{U} } \right.} \right. \\ & \quad \left. { + a_{24}^{U} ;min\left( {H_{1} \left( {\tilde{A}_{1}^{U} } \right),H_{1} \left( {\tilde{A}_{2}^{U} } \right)} \right),min\left( {H_{2} \left( {\tilde{A}_{1}^{U} } \right),H_{2} \left( {\tilde{A}_{2}^{U} } \right)} \right)} \right), \\ & \quad \left. {\left( {a_{11}^{L} + a_{21}^{L} ,a_{12}^{L} + a_{22}^{L} ,a_{13}^{L} + a_{23}^{L} ,a_{14}^{L} + a_{24}^{L} ;min\left( {H_{1} \left( {\tilde{A}_{1}^{L} } \right),H_{1} \left( {\tilde{A}_{2}^{L} } \right)} \right),min\left( {H_{2} \left( {\tilde{A}_{1}^{L} } \right),H_{2} \left( {\tilde{A}_{2}^{L} } \right)} \right)} \right)} \right) \\ \end{aligned}$$

5 $$\begin{aligned} \tilde{A}_{1} { \ominus }\tilde{A}_{2} & = \left( {\tilde{A}_{1}^{U} ,\tilde{A}_{1}^{L} } \right){ \ominus }\left( {\tilde{A}_{2}^{U} ,\tilde{A}_{2}^{L} } \right) = \left( {\left( {a_{11}^{U} - a_{24}^{U} ,a_{12}^{U} - a_{23}^{U} ,a_{13}^{U} - a_{22}^{U} ,a_{14}^{U} } \right.} \right. \\ & \quad \left. { - a_{21}^{U} ;min\left( {H_{1} \left( {\tilde{A}_{1}^{U} } \right),H_{1} \left( {\tilde{A}_{2}^{U} } \right)} \right),min\left( {H_{2} \left( {\tilde{A}_{1}^{U} } \right),H_{2} \left( {\tilde{A}_{2}^{U} } \right)} \right)} \right), \\ & \quad \left. {{ }\left( {a_{11}^{L} - a_{24}^{L} ,a_{12}^{L} - a_{23}^{L} ,a_{13}^{L} - a_{22}^{L} ,a_{14}^{L} - a_{21}^{L} ;min\left( {H_{1} \left( {\tilde{A}_{1}^{L} } \right),H_{1} \left( {\tilde{A}_{2}^{L} } \right)} \right),min\left( {H_{2} \left( {\tilde{A}_{1}^{L} } \right),H_{2} \left( {\tilde{A}_{2}^{L} } \right)} \right)} \right)} \right) \\ \end{aligned}$$

6 $$\begin{aligned} \tilde{A}_{1} \otimes \tilde{A}_{2} & = \left( {\tilde{A}_{1}^{U} ,\tilde{A}_{1}^{L} } \right) \otimes \left( {\tilde{A}_{2}^{U} ,\tilde{A}_{2}^{L} } \right) = \left( {\left( {a_{11}^{U} \times a_{21}^{U} ,a_{12}^{U} \times a_{22}^{U} ,a_{13}^{U} \times a_{23}^{U} ,a_{14}^{U} } \right.} \right. \\ & \quad \left. { \times a_{24}^{U} ;min\left( {H_{1} \left( {\tilde{A}_{1}^{U} } \right),H_{1} \left( {\tilde{A}_{2}^{U} } \right)} \right),min\left( {H_{2} \left( {\tilde{A}_{1}^{U} } \right),H_{2} \left( {\tilde{A}_{2}^{U} } \right)} \right)} \right), \\ & \quad \left. {\left( {a_{11}^{L} \times a_{21}^{L} ,a_{12}^{L} \times a_{22}^{L} ,a_{13}^{L} \times a_{23}^{L} ,a_{14}^{L} \times a_{24}^{L} ;min\left( {H_{1} \left( {\tilde{A}_{1}^{L} } \right),H_{1} \left( {\tilde{A}_{2}^{L} } \right)} \right),min\left( {H_{2} \left( {\tilde{A}_{1}^{L} } \right),H_{2} \left( {\tilde{A}_{2}^{L} } \right)} \right)} \right)} \right) \\ \end{aligned}$$

7 $$k\tilde{A}_{1} = \left( {k \times a_{11}^{U} ,k \times a_{12}^{U} ,k \times a_{13}^{U} ,k \times a_{14}^{U} ;H_{1} \left( {\tilde{A}_{1}^{U} } \right),H_{2} \left( {\tilde{A}_{1}^{U} } \right)} \right),\left( {k \times a_{11}^{L} ,k \times a_{12}^{L} ,k \times a_{13}^{L} ,k \times a_{14}^{L} ;H_{1} \left( {\tilde{A}_{1}^{L} } \right),H_{2} \left( {\tilde{A}_{1}^{L} } \right)} \right)$$

8 $$\frac{{\tilde{A}_{1} }}{k} = \left( {\frac{1}{k} \times a_{11}^{U} ,\frac{1}{k} \times a_{12}^{U} ,\frac{1}{k} \times a_{13}^{U} ,\frac{1}{k} \times a_{14}^{U} ;H_{1} \left( {\tilde{A}_{1}^{U} } \right),H_{2} \left( {\tilde{A}_{1}^{U} } \right)} \right),\left( {\frac{1}{k} \times a_{11}^{L} ,\frac{1}{k} \times a_{12}^{L} ,\frac{1}{k} \times a_{13}^{L} ,\frac{1}{k} \times a_{14}^{L} ;H_{1} \left( {\tilde{A}_{1}^{L} } \right),H_{2} \left( {\tilde{A}_{1}^{L} } \right)} \right)$$

### IT2F-DEMATEL

The first stage of hybrid modeling is the application of the DEMATEL method based on IT2 fuzzy sets to measure the relative importance of each Fintech-based determinant. The method provides comprehensive results by obtaining influence degrees of each criterion in addition to the weights of the factors. The first step of the DEMATEL method is to construct the direct-relation matrix. The average values provided by the decision-makers are used to construct the relation matrix. The normalization procedure is then employed in Eqs. (9)–(12) (Xu et al. 2020).

9 $$\tilde{Z} = \left[ {\begin{array}{*{20}c} 0 & {\tilde{z}_{12} } & \cdots & {} & \cdots & {\tilde{z}_{1n} } \\ {\tilde{z}_{21} } & 0 & \cdots & {} & \cdots & {\tilde{z}_{2n} } \\ \vdots & \vdots & \ddots & {} & \cdots & \cdots \\ \vdots & \vdots & \vdots & {} & \ddots & \vdots \\ {\tilde{z}_{n1} } & {\tilde{z}_{n2} } & \cdots & {} & \cdots & 0 \\ \end{array} } \right]$$

10 $$\tilde{Z} = \frac{{\tilde{Z}^{1} + \tilde{Z}^{2} + \tilde{Z}^{3} + \ldots \tilde{Z}^{n} }}{n}$$

11 $$\tilde{x}_{ij} = \frac{{\tilde{z}_{ij} }}{r} = \left( {\frac{{Z_{{\mathop {a_{ij} }\limits }} }}{r},\frac{{Z_{{b_{ij}^{^{\prime}} }} }}{r},\frac{{Z_{{c_{ij}^{^{\prime}} }} }}{r},\frac{{Z_{{d_{ij}^{^{\prime}} }} }}{r};H_{1} \left( {z_{ij}^{U} } \right),H_{2} \left( {z_{ij}^{U} } \right)} \right),\left( {\frac{{Z_{{e_{ij}^{^{\prime}} }} }}{r},\frac{{Z_{{f_{ij}^{^{\prime}} }} }}{r},\frac{{Z_{{g_{ij}^{^{\prime}} }} }}{r},\frac{{Z_{{h_{ij}^{^{\prime}} }} }}{r};H_{1} \left( {z_{ij}^{L} } \right),H_{2} \left( {z_{ij}^{L} } \right)} \right)$$

12 $$r = max\left( {max_{1 \le i \le n} \mathop \sum \limits_{j = 1}^{n} Z_{{d_{ij}^{^{\prime}} }} ,max_{1 \le i \le n} \mathop \sum \limits_{j = 1}^{n} Z_{{d_{ij}^{^{\prime}} }} } \right)$$

The second stage is to compute the total relation matrix using Eqs. (13)–(17) (Garg 2021).

13 $$X_{{\grave{a}}} = \left[ {\begin{array}{*{20}c} 0 & {a^{\prime}_{12} } & \cdots & {} & \cdots & {a^{\prime}_{1n} } \\ {a^{\prime}_{21} } & 0 & \cdots & {} & \cdots & {a^{\prime}_{2n} } \\ \vdots & \vdots & \ddots & {} & \cdots & \cdots \\ \vdots & \vdots & \vdots & {} & \ddots & \vdots \\ {a^{\prime}_{n1} } & {a^{\prime}_{n2} } & \cdots & {} & \cdots & 0 \\ \end{array} } \right], \ldots ,\,X_{\mathop h\limits } = \left[ {\begin{array}{*{20}c} 0 & {h^{\prime}_{12} } & \cdots & {} & \cdots & {h^{\prime}_{1n} } \\ {h^{\prime}_{21} } & 0 & \cdots & {} & \cdots & {h^{\prime}_{2n} } \\ \vdots & \vdots & \ddots & {} & \cdots & \cdots \\ \vdots & \vdots & \vdots & {} & \ddots & \vdots \\ {h^{\prime}_{n1} } & {h^{\prime}_{n2} } & \cdots & {} & \cdots & 0 \\ \end{array} } \right]$$

14 $$\tilde{T} = \mathop {\lim }\limits_{k \to \infty } \tilde{X} + \tilde{X}^{2} + \ldots + \tilde{X}^{k}$$

15 $$\tilde{T} = \left[ {\begin{array}{*{20}c} {\tilde{t}_{11} } & {\tilde{t}_{12} } & \cdots & {} & \cdots & {\tilde{t}_{1n} } \\ {\tilde{t}_{21} } & {\tilde{t}_{22} } & \cdots & {} & \cdots & {\tilde{t}_{2n} } \\ \vdots & \vdots & \ddots & {} & \cdots & \cdots \\ \vdots & \vdots & \vdots & {} & \ddots & \vdots \\ {\tilde{t}_{n1} } & {\tilde{t}_{n2} } & \cdots & {} & \cdots & {\tilde{t}_{nn} } \\ \end{array} } \right]$$

16 $$\tilde{t}_{ij} = \left( {a^{\prime\prime}_{ij} ,b^{\prime\prime}_{ij} ,c^{\prime\prime}_{ij} ,d^{\prime\prime}_{ij} ;H_{1} \left( {\tilde{t}_{ij}^{U} } \right),H_{2} \left( {\tilde{t}_{ij}^{U} } \right)} \right),\left( {e^{\prime\prime}_{ij} ,f^{\prime\prime}_{ij} ,g^{\prime\prime}_{ij} ,h^{\prime\prime}_{ij} ;H_{1} \left( {\tilde{t}_{ij}^{L} } \right),H_{2} \left( {\tilde{t}_{ij}^{L} } \right)} \right)$$

17 $$\left[ {a^{\prime\prime}_{ij} } \right] = X_{{\grave{a}}} \times \left( {I - X_{{\grave{a}}} } \right)^{ - 1} \,, \ldots ,\,\left[ {h^{\prime\prime}_{ij} } \right] = X_{{\grave{h}}} \times \left( {I - X_{{\grave{h}}} } \right)^{ - 1}$$

The defuzzified values of the total relation matrix are then calculated. The impact and degree of relation among each criterion is measured using Eqs. (18) – (21) (Zhang et al. 2020a, b).

18 $$Def_{T} = \frac{{\frac{{\left( {u_{U} - l_{U} } \right) + \left( {{\upbeta }_{U} \times m_{1U} - l_{U} } \right) + \left( {{\upalpha }_{U} \times m_{2U} - l_{U} } \right)}}{4} + l_{U} + \left[ {\frac{{\left( {u_{L} - l_{L} } \right) + \left( {{\upbeta }_{L} \times m_{1L} - l_{L} } \right) + \left( {{\upalpha }_{L} \times m_{2L} - l_{L} } \right)}}{4} + l_{L} } \right]}}{2}$$

19 $$Def_{T} = T = \left[ {t_{ij} } \right]_{n \times n} ,\,i,j = 1,2, \ldots ,n$$

20 $$\tilde{D}_{i}^{def} = r = \left[ {\mathop \sum \limits_{j = 1}^{n} t_{ij} } \right]_{n \times 1} = \left( {r_{i} } \right)_{n \times 1} = \left( {r_{1} , \ldots ,r_{i} , \ldots ,r_{n} } \right)$$

21 $$\tilde{R}_{i}^{def} = y = \left[ {\mathop \sum \limits_{i = 1}^{n} t_{ij} } \right]_{1 \times n}^{^{\prime}} = \left( {y_{j} } \right)_{1 \times n}^{^{\prime}} = \left( {y_{1} , \ldots ,y_{i} , \ldots ,y_{n} } \right)$$

where $\tilde{D}_{i}^{def}$ and $\tilde{R}_{i}^{def}$ represent the sum of all vector rows and columns, respectively. The influence degrees are presented as $\left( {\tilde{D}_{i} - \tilde{R}_{i} } \right)^{def}$ and the relative importance is computed by $\left( {\tilde{D}_{i} + \tilde{R}_{i} } \right)^{def}$ (Wang et al. 2020).

### IT2F-TOPSIS

The integrated decision-making model continues with an application of the IT2 fuzzy TOPSIS method. The method handles negative and positive ideal solutions in the multi-criteria decision-making problem and measures distances from the ideal solution (Rani et al. 2020).

The first step of TOPSIS is to construct the decision matrix. Average scores of the decision-makers are used to contract the fuzzy decision matrix in Eqs. (22)–(23) (Dhiman and Deb 2020).

22 $$X_{{ij}} = \begin{array}{*{20}l} {} & {\begin{array}{*{20}l} {C_{1} } \hfill & {C_{2} } \hfill & {C_{3} } \hfill & \ldots \hfill & {C_{n} } \hfill \\ \end{array} } \\ {\begin{array}{*{20}l} {A_{1} } \\ {A_{2} } \\ {A_{3} } \\ \vdots \\ {A_{m} } \\ \end{array} } & {\left[ {\begin{array}{*{20}l} {x_{{11}} } & {x_{{12}} } & {x_{{13}} } & \ldots & {x_{{1n}} } \\ {x_{{21}} } & {x_{{22}} } & {x_{{23}} } & \ldots & {x_{{2n}} } \\ {x_{{31}} } & {x_{{32}} } & {x_{{33}} } & \ldots & {x_{{3n}} } \\ \vdots & \vdots & \ddots & \ldots & \vdots \\ {x_{{m1}} } & {x_{{m2}} } & {x_{{m3}} } & \ldots & {x_{{mn}} } \\ \end{array} } \right]} \\ \end{array}$$

23 $$X_{ij} = \frac{1}{K}\left[ {\mathop \sum \limits_{e = 1}^{n} X_{ij}^{e} } \right], \quad i = 1,2,3, \ldots ,m$$

where the aggregated fuzzy values are X_ij_, and the number of decision-makers is defined as *K*.

The second step is to compute the defuzzified values of the decision matrix following Chen and Lee (2010) in Eqs. (24)–(27) (Rouyendegh et al. 2020).

24 $$\begin{aligned} Def\left( {x_{ij} } \right) & = Rank(\tilde{x}_{ij} )_{m \times n} = M_{1} \left( {\tilde{A}_{i}^{U} } \right) + M_{1} \left( {\tilde{A}_{i}^{L} } \right) + M_{2} \left( {\tilde{A}_{i}^{U} } \right) + M_{2} \left( {\tilde{A}_{i}^{L} } \right) + M_{3} \left( {\tilde{A}_{i}^{U} } \right) \\ & \quad + M_{3} \left( {\tilde{A}_{i}^{L} } \right) - \frac{1}{4}\left( {S_{1} \left( {\tilde{A}_{i}^{U} } \right) + S_{1} \left( {\tilde{A}_{i}^{L} } \right) + S_{2} \left( {\tilde{A}_{i}^{U} } \right) + S_{2} \left( {\tilde{A}_{i}^{L} } \right) + S_{3} \left( {\tilde{A}_{i}^{U} } \right) + S_{3} \left( {\tilde{A}_{i}^{L} } \right) + S_{4} \left( {\tilde{A}_{i}^{U} } \right) + S_{4} \left( {\tilde{A}_{i}^{L} } \right)} \right) \\ & \quad + H_{1} \left( {\tilde{A}_{i}^{U} } \right) + H_{1} \left( {\tilde{A}_{i}^{L} } \right) + H_{2} \left( {\tilde{A}_{i}^{U} } \right) + H_{2} \left( {\tilde{A}_{i}^{L} } \right) \\ \end{aligned}$$

25 $$M_{p} \left( {\tilde{A}_{i}^{j} } \right) = \left( {a_{ip}^{j} + a_{{i\left( {p + 1} \right)}}^{j} } \right)/2$$

where $M_{p} \left( {\tilde{A}_{i}^{j} } \right)$ is the average of $a_{ip}^{j}$ and $a_{{i\left( {p + 1} \right)}}^{j}$, $1 \le p \le 3$,

26 $$S_{q} \left( {\tilde{A}_{i}^{j} } \right) = \sqrt {\frac{1}{2}\mathop \sum \limits_{k = q}^{q + 1} \left( {a_{ik}^{j} - \frac{1}{2}\mathop \sum \limits_{k = q}^{q + 1} a_{ik}^{j} } \right)^{2} }$$

where $S_{q} \left( {\tilde{A}_{i}^{j} } \right)$ is the standard deviation of $a_{iq}^{j}$ and $a_{{i\left( {q + 1} \right)}}^{j}$, $1 \le q \le 3$,

27 $$S_{4} \left( {\tilde{A}_{i}^{j} } \right) = \sqrt {\frac{1}{4}\mathop \sum \limits_{k = 1}^{4} \left( {a_{ik}^{j} - \frac{1}{4}\mathop \sum \limits_{k = 1}^{4} a_{ik}^{j} } \right)^{2} }$$

$H_{p} \left( {\tilde{A}_{i}^{j} } \right)$ is the membership value of $a_{{i\left( {p + 1} \right)}}^{j}$ in the trapezoidal membership function $\tilde{A}_{i}^{j}$, $1 \le p \le 2$, $j \in \left\{ {U,L} \right\}$ $1 \le i \le n$. The third step of the TOPSIS method is to compute the closeness coefficient (CC_i_) in Eqs. (28)–(32) (Petrovic and Kankaras 2020).

28 $$A^{ + } = \left\{ {v_{1}^{ + } , \ldots v_{n}^{ + } } \right\} = \left\{ {\left( {{}_{i}^{max} v_{ij} ,j \in J} \right)\left( {{}_{i}^{min} v_{ij} ,j \in \grave{J}} \right)} \right\}, i = 1,2, \ldots m$$

29 $$A^{ - } = \left\{ {v_{1}^{ - } , \ldots v_{n}^{ - } } \right\} = \left\{ {\left( {{}_{i}^{min} v_{ij} ,j \in J} \right)\left( {{}_{i}^{max} v_{ij} ,j \in \grave{J}} \right)} \right\}, i = 1,2, \ldots m$$

Within this context, $v_{ij}$ is the weights of the factors. $A^{ + }$ defines the positive ideal value set whereas $A^{ - }$ provides information about the negative ideal value set (Noureddine and Ristic 2019). However, *J* is associated with the benefit criteria and $\grave{J}$ with cost criteria (Zienba et al., 2020).

30 $$D_{i}^{ + } = \sqrt {\mathop \sum \limits_{i = 1}^{m} (v_{i} - A_{i}^{ + } )^{2} }$$

31 $$D_{i}^{ - } = \sqrt {\mathop \sum \limits_{i = 1}^{m} (v_{i} - A_{i}^{ - } )^{2} }$$

32 $$CC_{i} = \frac{{D_{i}^{ - } }}{{D_{i}^{ + } + D_{i}^{ - } }}$$

### IT2F-VIKOR

The VIKOR methodology is also considered to rank the alternatives. In the first step, a fuzzy decision matrix is generated with the help of the same procedure of IT2 fuzzy TOPSIS. Then, the fuzzy best value $\tilde{f}_{j}^{*}$ and fuzzy worst value $\tilde{f}_{j}^{ - }$ are calculated using Eq. (33).

33 $$\tilde{f}_{J}^{*} = \mathop {max}\limits_{i} \tilde{x}_{ij} ,\,{\text{and}}\,\tilde{f}_{j}^{ - } = \mathop {min}\limits_{i} \tilde{x}_{ij} ,$$

In the following step, the mean group utility and maximal regret are computed as in Eqs. (34) and (35), respectively.

34 $$\tilde{S}_{i} = \mathop \sum \limits_{i = 1}^{n} \tilde{w}_{j} \frac{{\left( {\left| {\tilde{f}_{j}^{*} - \tilde{x}_{ij} } \right|} \right)}}{{\left( {\left| {\tilde{f}_{j}^{*} - \tilde{f}_{j}^{ - } } \right|} \right)}}$$

35 $$\tilde{R}_{i} = \max_{j} \left[ {\tilde{w}_{j} \frac{{\left( {\left| {\tilde{f}_{j}^{*} - \tilde{x}_{ij} } \right|} \right)}}{{\left( {\left| {\tilde{f}_{j}^{*} - \tilde{f}_{j}^{ - } } \right|} \right)}}} \right]$$

In these equations, $\tilde{w}_{j}$ represents the fuzzy weights, while $\tilde{S}_{i}$ is Ai regarding all criteria calculated by the total of the distance for the fuzzy best value. On the other side, $\tilde{R}_{i}$ is Ai with respect to the *j*-th criterion, which can be calculated by the maximum distance of the fuzzy best value. Next, the value of $\tilde{Q}_{i}$ is calculated using Eq. (36).

36 $$\tilde{Q}_{i} = v\left( {\tilde{S}_{i} - \tilde{S}^{*} } \right)/\left( {\tilde{S}^{ - } - \tilde{S}^{*} } \right) + \left( {1 - v} \right)\left( {\tilde{R}_{i} - \tilde{R}^{*} } \right)/\left( {\tilde{R}^{ - } - \tilde{R}^{*} } \right)$$

In this framework, *v* identifies the weight of the strategy of maximum group utility. Moreover, 1 – *v* shows the weight of the individual regret. In this study, *v* is accepted as 0.5. In the final stage, the values of *S*, *R*, and *Q* are calculated, which are used to rank the alternatives. With respect to checking the final ranks, two conditions should be satisfied. The first condition is related to the acceptable advantage shown in Eq. (37).

37 $$Q\left( {A^{\left( 2 \right)} } \right) - Q\left( {A^{\left( 1 \right)} } \right) \ge 1/\left( {j - 1} \right)$$

The second condition focused on the acceptable stability in the decision-making process. When one of the conditions is not satisfied, different conditions are taken into consideration. If the second condition is not satisfied, then it means the solution is composed of alternatives $A^{\left( 1 \right)}$ and $A^{\left( 2 \right)}$. If the first condition is not satisfied, then the alternatives $A^{\left( 1 \right)}$, $A^{\left( 2 \right)}$…, $A^{\left( M \right)}$ are used.

## Empirical findings

This section presents our empirical findings of weights and ranking of suggested alternatives. First, we calculated the initial direct-relation matrix for the criteria (Table 11 of “Appendix B”). The total relation matrix and the defuzzified values are presented in Table 12 of “Appendix B”. Table 6 below reports the weighting results for the dimensions and criteria, which are calculated by studying the values of $\tilde{D}_{i}^{def}$, $\tilde{R}_{i}^{def}$, and $\left( {\tilde{D}_{i} + \tilde{R}_{i} } \right)^{def}$ from the defuzzified matrix.

**Table 6:** Weighting results of criteria and dimensions. *Source*: Author’s calculations

Criteria	$\tilde{D}_{i}^{def}$	$\tilde{R}_{i}^{def}$	$\left( {\tilde{D}_{i} + \tilde{R}_{i} } \right)^{def}$	$\left( {\tilde{D}_{i} - \tilde{R}_{i} } \right)^{def}$	Criterion weights	Dimension weights
C1	2.10	1.02	3.12	1.07	0.172	Financial (0.48)
C2	1.11	1.52	2.63	− 0.40	0.145	
C3	1.24	1.71	2.94	− 0.47	0.163	
C4	1.83	1.26	3.09	0.57	0.171	Non-financial (0.52)
C5	1.91	1.64	3.55	0.27	0.196	
C6	0.87	1.91	2.78	− 1.04	0.153	

Our findings in Table 6 demonstrate that competitive advantage (criterion 5) is the most important factor among the Fintech-based determinants, while sales volume (criterion 2) is identified as having the weakest importance. Weighting results of the criteria can be listed as competitive advantage (criterion 5), cost management (criterion 1), customer satisfaction (criterion 4), increase in market value (criterion 3), organizational efficiency (criterion 6), and sales volume (criterion 2), respectively. The sum scores of each dimension suggests that non-financial factors are more important than the financial factors defining the Fintech-based determinants.

**Fig. 3:**
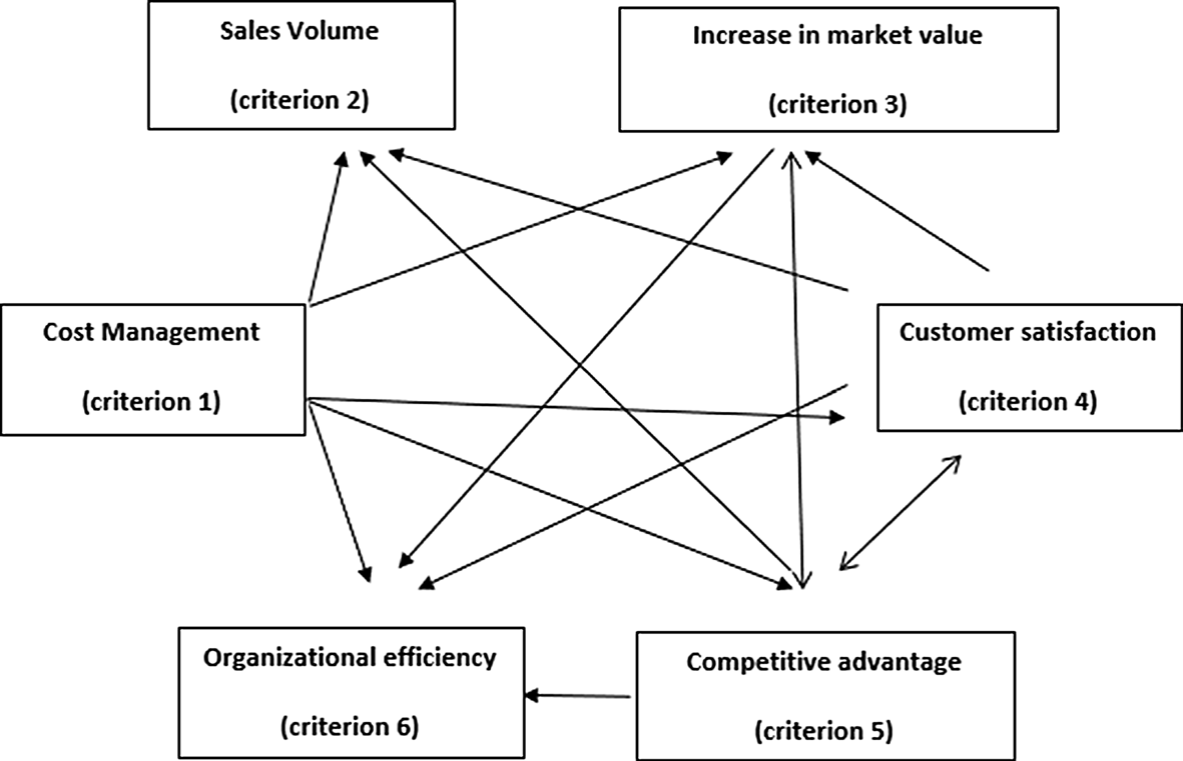
Impact and relation map of fintech-based determinants. *Source*: Created by the authors

The impact-relation directions among the six criteria are illustrated in Fig. 3. According to the findings, sales volume (criterion 2) has no impact on the other criteria as none affect cost management (criterion 1). Cost management (criterion 1) has the strongest influence on the other criteria, while increase in market value (criterion 3) is the second weakest factor following sales volume.

The final step of the TOPSIS method is to calculate the ranking scores with the help of Eqs. (20)–(23). In this respect, values of D^+^, D^−^, and closeness coefficient are computed. The values of the closeness coefficient are listed in decreasing order. The ranking results of the suggested alternatives are reported in Table 7.

**Table 7:** Closeness coefficient and ranking results for the alternatives. *Source*: Author’s calculations

Alternatives	D^+^	D^−^	Closeness coefficient	Ranking
Money transferring (alternative 1)	0.18	0.99	0.85	2
Payments (alternative 2)	0.03	1.12	0.97	1
Savings (alternative 3)	1.14	0.00	0.00	5
Budgeting (alternative 4)	0.99	0.18	0.16	4
Borrowings (alternative 5)	0.67	0.51	0.43	3

We also checked our results for consistency by employing a sensitivity analysis with six cases. The weights of the criteria are changed consecutively, and the rankings are reported with the changed weighting results. The findings are presented in Table 8.

**Table 8:** Ranking results with sensitivity analysis

Alternatives	Case 1	Case 2	Case 3	Case 4	Case 5	Case 6
Money transferring (alternative 1)	2	2	2	2	2	2
Payments (alternative 2)	1	1	1	1	1	1
Savings (alternative 3)	5	5	5	5	5	5
Budgeting (alternative 4)	4	4	4	3	3	4
Borrowings (alternative 5)	3	3	3	4	4	3

**Fig. 4:**
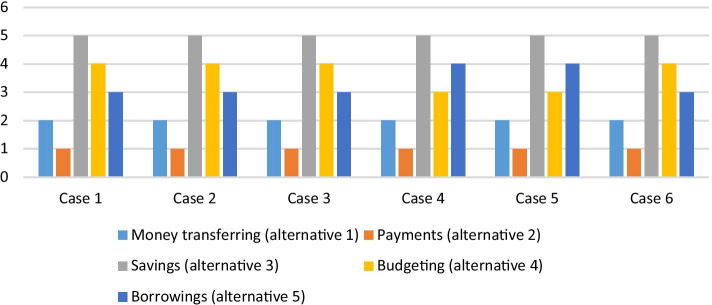
Sensitivity analysis results

The results of the sensitivity analysis are introduced in Fig. 4.

Sensitivity analysis results illustrate that the weighting priorities of the criteria are almost consistent for all cases. Moreover, a comparative analysis is applied for robustness check with the help of the IT2F-VIKOR method. The ranking results of both methods are presented in Table 9.

**Table 9:** Comparative ranking results

Alternatives	IT2F-TOPSIS	IT2F-VIKOR
Money transferring (alternative 1)	2	2
Payments (alternative 2)	1	1
Savings (alternative 3)	5	4
Budgeting (alternative 4)	4	5
Borrowings (alternative 5)	3	3

**Fig. 5:**
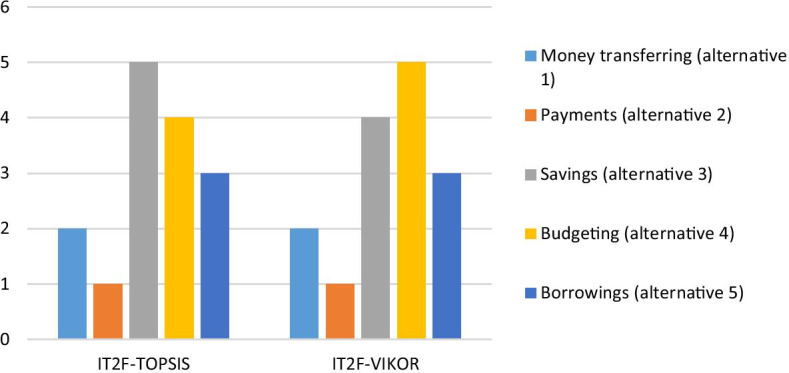
Comparative analysis results

Furthermore, Fig. 5 illustrates the comparative evaluation results.

The results for IT2F-TOPSIS and the IT2F-VIKOR are similar. This is clear evidence that the proposed hybrid model is applicable for the extensions of TOPSIS and VIKOR with the IT2 fuzzy sets.

The ranking results are listed as payments (alternative 2), money transferring (alternative 1), borrowings (alternative 5), budgeting (alternative 4), and savings (alternative 3). Accordingly, the overall results reveal that payments (alternative 2) are the strongest Fintech-based investment alternative, while savings (alternative 3) is the weakest for European banking services. Our empirical findings suggest that European banks should mainly focus on payment alternatives for Fintech-based investments to attract more customers. Consistent with Guo and Liang (2016) and Du et al. (2019), this may suggest that bank customers prefer easily accessible and user-friendly payment systems.

Furthermore, consistent with Yao et al. (2018a, b) and Ramos-de-Luna et al. (2016), our findings illustrate that money transferring is another important Fintech-based investment alternative. Banks play a key role in money transferring systems and Fintech-based investments can help banks to decrease their operational costs. In addition, this is expected to increase customer satisfaction in terms of competitive advantage and in return increase banks’ sales volume.

## Conclusion and future research

This study evaluates Fintech-based investments of European banking services. To achieve this objective, we select three financial and three non-financial criteria based on the relevant literature and define five Fintech-based investment alternatives. Our empirical analysis employs the IT2 fuzzy DEMATEL method to weight the criteria and the IT2 fuzzy TOPSIS method to rank the investment alternatives. A consistency check is accomplished by applying the VIKOR method. Furthermore, a sensitivity analysis is conducted for six individual cases to check the coherency and reliability of the empirical findings.

Results of the IT2 fuzzy DEMATEL approach indicate that “competitive advantage” is the most important factor among the Fintech-based determinants while “sales volume” has the weakest importance in the criterion set. Moreover, non-financial factors are found to be more important than financial factors in defining the Fintech-based determinants. Looking at the impact and relations map, we observe that “cost management” is the most influencing criterion and “sales volume” does not have an impact on others. Moreover, the results of IT2 fuzzy TOPSIS suggest payments and money transferring as the most important Fintech investment alternatives. Based on our empirical findings, we first suggest that European banks should mainly focus on payment alternatives for Fintech investments to attract customers’ attention and achieve effective collection of receivables. Second, Fintech investments in money transferring could help banks to decrease their costs, which is expected to have a positive influence on their sales volume. Our findings are consistent with studies such as Koomson and Ibrahim (2018), Azemi et al. (2019), Asamoah et al. (2020), Mensah et al. (2020), Gupta et al. (2019), Stulz (2019), and Yao et al. (2018a, b).

Moreover, spillover effects should also be taken into consideration while generating appropriate strategies to improve Fintech investments. Due to globalization, there has been significant expansion in financial markets (Jahmane and Gaies 2020). Fan et al. (2020) argued that this resulted in an increased economic and financial commitment of countries to each other. This is an indication that individual countries’ economies become more sensitive to problems experienced in other countries (Yin et al. 2020). National policies may not be very effective in certain situations such as Fintech investments. The results obtained herein are valid assuming that there are no serious global problems (Hofmann and Sertori 2020).

The main limitation of this study is that it focuses solely on the important points of Fintech-based investments and there are no industrial applications. We suggest a case evaluation in the banking sector for future research. Focusing on one country can help to provide direct recommendations to improve the financial system of that specific country. In addition, from the methodology side, empirical findings can be compared with other similar methods to understand the differences between quantitative data and expert opinions.

## Data Availability

All data generated or analyzed during this study are included in this article.

## Appendix A

See Table 10.

**Table 10:** Evaluation scales for the criteria and alternatives. *Source*: Adapted from Chen and Lee (2010), Baykasoğlu and Gölcük (2017) and Dincer and Yuksel (2019)

Alternative evaluations	Criterion evaluations	Interval type 2 fuzzy numbers
Very poor (VP)	Very low (VL)	((0, 0, 0, 0.1; 1, 1), (0, 0, 0, 0.05; 0.9, 0.9))
Poor (P)	Low (L)	((0, 0.1, 0.1, 0.3; 1, 1), (0.05, 0.1, 0.1, 0.2; 0.9, 0.9))
Medium poor (MP)	Medium low (ML)	((0.1, 0.3, 0.3, 0.5; 1, 1), (0.2, 0.3, 0.3, 0.4; 0.9, 0.9))
Fair (F)	Medium (M)	((0.3, 0.5, 0.5, 0.7; 1, 1), (0.4, 0.5, 0.5, 0.6; 0.9, 0.9))
Good (G)	Medium high (MH)	((0.5, 0.7, 0.7, 0.9; 1, 1), (0.6, 0.7, 0.7, 0.8; 0.9, 0.9))
Very good (VG)	High (H)	((0.7, 0.9, 0.9, 1; 1, 1), (0.8, 0.9, 0.9, 0.95; 0.9, 0.9))
Best (B)	Very high (VH)	((0.9, 1, 1, 1; 1, 1), (0.95, 1, 1, 1; 0.9, 0.9))

## Appendix B

See Tables 11 and 12.

**Table 11:** Initial direct relation matrix. *Source*: Author’s own table

	C1	C2	C3	C4	C5	C6
C1	((0, 0, 0, 0; 1, 1), (0, 0, 0, 0; 0.90, 0.90))	((0.63, 0.83, 0.83, 0.97; 1, 1), (0.73, 0.83, 0.83, 0.90; 0.90, 0.90))	((0.70, 0.90, 0.90, 1.00; 1, 1), (0.80, 0.90, 0.90, 0.95; 0.90, 0.90))	((0.43, 0.63, 0.63, 0.83; 1, 1), (0.53, 0.63, 0.63, 0.73; 0.90, 0.90))	((0.77, 0.93, 0.93, 1.00; 1, 1), (0.85, 0.93, 0.93, 0.97; 0.90, 0.90))	((0.83, 0.97, 0.97, 1.00; 1, 1), (0.90, 0.97, 0.97, 0.98; 0.90, 0.90))
C2	((0.23, 0.43, 0.43, 0.63; 1, 1), (0.33, 0.43, 0.43, 0.53; 0.90, 0.90))	((0, 0, 0, 0; 1, 1), (0, 0, 0, 0; 0.90, 0.90))	((0.30, 0.50, 0.50, 0.70; 1, 1), (0.40, 0.50, 0.50, 0.60; 0.90, 0.90))	((0.23, 0.43, 0.43, 0.63; 1, 1), (0.33, 0.43, 0.43, 0.53; 0.90, 0.90))	((0.10, 0.30, 0.30, 0.50; 1, 1), (0.20, 0.30, 0.30, 0.40; 0.90, 0.90))	((0.07, 0.23, 0.23, 0.43; 1, 1), (0.15, 0.23, 0.23, 0.33; 0.90, 0.90))
C3	((0.03, 0.13, 0.13, 0.27; 1, 1), (0.08, 0.13, 0.13, 0.20; 0.90, 0.90))	((0.30, 0.50, 0.50, 0.70; 1, 1), (0.40, 0.50, 0.50, 0.60; 0.90, 0.90))	((0, 0, 0, 0; 1, 1), (0, 0, 0, 0; 0.90, 0.90))	((0.17, 0.37, 0.37, 0.57; 1, 1), (0.27, 0.37, 0.37, 0.47; 0.90, 0.90))	((0.43, 0.63, 0.63, 0.83; 1, 1), (0.53, 0.63, 0.63, 0.73; 0.90, 0.90))	((0.50, 0.70, 0.70, 0.87; 1, 1), (0.60, 0.70, 0.70, 0.78; 0.90, 0.90))
C4	((0.30, 0.50, 0.50, 0.70; 1, 1), (0.40, 0.50, 0.50, 0.60; 0.90, 0.90))	((0.37, 0.57, 0.57, 0.77; 1, 1), (0.47, 0.57, 0.57, 0.67; 0.90, 0.90))	((0.43, 0.63, 0.63, 0.83; 1, 1), (0.53, 0.63, 0.63, 0.73; 0.90, 0.90))	((0, 0, 0, 0; 1, 1), (0, 0, 0, 0; 0.90, 0.90))	((0.77, 0.93, 0.93, 1.00; 1, 1), (0.85, 0.93, 0.93, 0.97; 0.90, 0.90))	((0.83, 0.97, 0.97, 1.00; 1, 1), (0.90, 0.97, 0.97, 0.98; 0.90, 0.90))
C5	((0.37, 0.57, 0.57, 0.77; 1, 1), (0.47, 0.57, 0.57, 0.67; 0.90, 0.90))	((0.57, 0.77, 0.77, 0.93; 1, 1), (0.67, 0.77, 0.77, 0.85; 0.90, 0.90))	((0.77, 0.93, 0.93, 1.00; 1, 1), (0.85, 0.93, 0.93, 0.97; 0.90, 0.90))	((0.43, 0.63, 0.63, 0.83; 1, 1), (0.53, 0.63, 0.63, 0.73; 0.90, 0.90))	((0, 0, 0, 0; 1, 1), (0, 0, 0, 0; 0.90, 0.90))	((0.83, 0.97, 0.97, 1.00; 1, 1), (0.90, 0.97, 0.97, 0.98; 0.90, 0.90))
C6	((0.03, 0.17, 0.17, 0.37; 1, 1), (0.10, 0.17, 0.17, 0.27; 0.90, 0.90))	((0.07, 0.23, 0.23, 0.43; 1, 1), (0.15, 0.23, 0.23, 0.33; 0.90, 0.90))	((0.17, 0.37, 0.37, 0.57; 1, 1), (0.27, 0.37, 0.37, 0.47; 0.90, 0.90))	((0.07, 0.23, 0.23, 0.43; 1, 1), (0.15, 0.23, 0.23, 0.33; 0.90, 0.90))	((0.23, 0.43, 0.43, 0.63; 1, 1), (0.33, 0.43, 0.43, 0.53; 0.90, 0.90))	((0, 0, 0, 0; 1, 1), (0, 0, 0, 0; 0.90, 0.90))

**Table 12:** Defuzzified total relation matrix. *Source*: Author’s own table

	C1	C2	C3	C4	C5	C6
C1	0.16	0.37	0.41	0.30	0.40	0.45
C2	0.17	0.13	0.23	0.19	0.20	0.20
C3	0.13	0.23	0.15	0.18	0.26	0.29
C4	0.23	0.30	0.34	0.17	0.37	0.42
C5	0.24	0.34	0.39	0.29	0.22	0.42
C6	0.10	0.15	0.18	0.13	0.18	0.12

## Appendix C

See Tables 13, 14 and 15.

**Table 13:** Decision matrix. *Source*: Author’s own table

	A1	A2	A3	A4	A5
C1	((0.70, 0.87, 0.87, 0.97; 1, 1), (0.78, 0.87, 0.87, 0.92; 0.90, 0.90))	((0.83, 0.97, 0.97, 1.00; 1, 1), (0.90, 0.97, 0.97, 0.98; 0.90, 0.90))	((0.37, 0.57, 0.57, 0.77; 1, 1), (0.47, 0.57, 0.57, 0.67; 0.90, 0.90))	((0.43, 0.63, 0.63, 0.83; 1, 1), (0.53, 0.63, 0.63, 0.73; 0.90, 0.90))	((0.5, 0.7, 0.7, 0.90; 1, 1), (0.6, 0.7, 0.7, 0.80; 0.90, 0.90))
C2	((0.63, 0.83, 0.83, 0.97; 1, 1), (0.73, 0.83, 0.83, 0.90; 0.90, 0.90))	((0.77, 0.93, 0.93, 1.00; 1.00, 1.00), (0.85, 0.93, 0.93, 0.97; 0.90, 0.90))	((0.17, 0.37, 0.37, 0.57; 1.00, 1.00), (0.27, 0.37, 0.37, 0.47; 0.90, 0.90))	((0.23, 0.43, 0.43, 0.63; 1.00, 1.00), (0.33, 0.43, 0.43, 0.53; 0.90, 0.90))	((0.37, 0.57, 0.57, 0.77; 1, 1), (0.47, 0.57, 0.57, 0.67; 0.90, 0.90))
C3	((0.70, 0.87, 0.87, 0.97; 1, 1), (0.78, 0.87, 0.87, 0.92; 0.90, 0.90))	((0.77, 0.93, 0.93, 1.00; 1.00, 1.00), (0.85, 0.93, 0.93, 0.97; 0.90, 0.90))	((0.30, 0.50, 0.50, 0.70; 1.00, 1.00), (0.40, 0.50, 0.50, 0.60; 0.90, 0.90))	((0.43, 0.63, 0.63, 0.83; 1, 1), (0.53, 0.63, 0.63, 0.73; 0.90, 0.90))	((0.57, 0.77, 0.77, 0.93; 1, 1), (0.67, 0.77, 0.77, 0.85; 0.90, 0.90))
C4	((0.77, 0.93, 0.93, 1.00; 1.00, 1.00), (0.85, 0.93, 0.93, 0.97; 0.90, 0.90))	((0.83, 0.97, 0.97, 1.00; 1, 1), (0.90, 0.97, 0.97, 0.98; 0.90, 0.90))	((0.37, 0.57, 0.57, 0.77; 1, 1), (0.47, 0.57, 0.57, 0.67; 0.90, 0.90))	((0.43, 0.63, 0.63, 0.83; 1, 1), (0.53, 0.63, 0.63, 0.73; 0.90, 0.90))	((0.63, 0.83, 0.83, 0.97; 1, 1), (0.73, 0.83, 0.83, 0.90; 0.90, 0.90))
C5	((0.70, 0.90, 0.90, 1.00; 1, 1), (0.80, 0.90, 0.90, 0.95; 0.90, 0.90))	((0.83, 0.97, 0.97, 1.00; 1, 1), (0.90, 0.97, 0.97, 0.98; 0.90, 0.90))	((0.30, 0.50, 0.50, 0.70; 1.00, 1.00), (0.40, 0.50, 0.50, 0.60; 0.90, 0.90))	((0.30, 0.50, 0.50, 0.70; 1.00, 1.00), (0.40, 0.50, 0.50, 0.60; 0.90, 0.90))	((0.5, 0.7, 0.7, 0.90; 1, 1), (0.6, 0.7, 0.7, 0.80; 0.90, 0.90))
C6	((0.83, 0.97, 0.97, 1.00; 1, 1), (0.90, 0.97, 0.97, 0.98; 0.90, 0.90))	((0.77, 0.93, 0.93, 1.00; 1.00, 1.00), (0.85, 0.93, 0.93, 0.97; 0.90, 0.90))	((0.23, 0.43, 0.43, 0.63; 1.00, 1.00), (0.33, 0.43, 0.43, 0.53; 0.90, 0.90))	((0.30, 0.50, 0.50, 0.70; 1.00, 1.00), (0.40, 0.50, 0.50, 0.60; 0.90, 0.90))	((0.37, 0.57, 0.57, 0.77; 1, 1), (0.47, 0.57, 0.57, 0.67; 0.90, 0.90))

**Table 14:** Defuzzified decision matrix. *Source*: Author’s own table

	A1	A2	A3	A4	A5
C1	8.86	9.47	7.07	7.47	7.87
C2	8.64	9.25	5.87	6.27	7.07
C3	8.86	9.25	6.67	7.47	8.26
C4	9.25	9.47	7.07	7.47	8.64
C5	9.03	9.47	6.67	6.67	7.87
C6	9.47	9.25	6.27	6.67	7.07

**Table 15:** Weighted decision matrix. *Source*: Author’s own table

	A1	A2	A3	A4	A5
C1	1.53	1.63	1.22	1.29	1.36
C2	1.25	1.34	0.85	0.91	1.03
C3	1.44	1.50	1.08	1.21	1.34
C4	1.58	1.62	1.21	1.28	1.48
C5	1.77	1.86	1.31	1.31	1.54
C6	1.45	1.42	0.96	1.02	1.08
